# Editorial: Cognitive mechanisms of visual attention, working memory, emotion, and their interactions

**DOI:** 10.3389/fnins.2023.1259002

**Published:** 2023-07-26

**Authors:** Qianru Xu, Qiang Liu, Chaoxiong Ye

**Affiliations:** ^1^Institute of Brain and Psychological Sciences, Sichuan Normal University, Chengdu, China; ^2^Center for Machine Vision and Signal Analysis (CMVS), University of Oulu, Oulu, Finland; ^3^Department of Psychology, Faculty of Education and Psychology, University of Jyväskylä, Jyväskylä, Finland; ^4^Faculty of Social Sciences, Tampere University, Tampere, Finland

**Keywords:** visual attention, working memory, emotion, cognitive process, cognitive neuroscience

We, as human beings, inhabit a visually rich world that necessitates the cooperation of various cognitive systems to function and adapt effectively within this environment. Notably, visual attention plays a crucial role in selectively focusing on specific information and further processing it, facilitating efficient information processing. Working memory (WM) enables us to temporarily store and manipulate the information captured by attention, allowing us to handle more complex tasks. Furthermore, our emotional system actively participates in both attention and WM processes, by exerting influence and bias on them. Undoubtedly, the interconnectedness of these three systems is of great importance. However, it is common for researchers to investigate these cognitive processes separately, which ultimately leads to a limited understanding of the underlying neural basis and the interactions among them. Thus, the objective of this Research Topic collection is to bring together researchers from these three areas, with a particular focus on studies exploring the underlying interactions and mechanisms between these functions and how they mutually influence each other.

Fundamentally, WM provides a framework of prior knowledge and expectations, guiding attention and aiding in the understanding of incoming stimuli. For instance, Chen Z. et al. investigated whether the memory-driven factor or the cue-driven factor from WM representation affects visual search. The authors discovered that when controlling for WM representation as an additional variable, the memory and target match condition (i.e., the positive cue) had a lesser impact and even caused some impairment in subsequent visual search compared to the memory and non-target match condition. Therefore, the authors suggest that there are distinct components of target and distractor templates in our WM system, and the positive cueing effect observed in previous studies could primarily be driven by automatic WM guidance rather than the effect from the predictive cue itself. By conducting an Electroencephalograph (EEG) study during Shepard-Metzler's mental rotation task (SMT), Anomal et al. examined the link between cortical activation and visuospatial SMT performance in adolescents. The researchers measured WM skills and other dimensions of IQ scores using intelligence scales. The findings revealed a negative correlation between WM scores and alpha activity in the frontal cortex, particularly during the challenging task condition. This suggests the crucial role of the frontal lobe in both WM abilities and SMT performance.

Our WM has limited capacity, but we often employ various strategies to accommodate more items. For example, Ren et al. investigated whether memory performance for identical objects improves due to the strengthened associations between them. They conducted three-color recall tasks and discovered evidence of a facilitation effect of identical objects in WM. However, they also demonstrated that this facilitation effect was influenced by the location information of the objects, which requires further investigation. In addition to the self-employed strategies, WM can also be influenced by various other external factors. Through a systematic review of previous studies, Zhu et al. discovered that physical activity had a small yet significant positive impact on visual-spatial working memory (VSWM) in healthy individuals. This impact was particularly notable in children and seniors after engaging in long-term exercise. As a result, the researchers recommended specific exercise intervention programs that could be considered for these age groups. In turn, WM training has the potential to enhance other cognitive functions. Wang et al. conducted a study that provided EEG evidence supporting the beneficial effects of WM training on enhancing fluid intelligence in children. The researchers specifically identified the predictive role of response inhibition ability in this improvement, offering both theoretical understanding and practical implications for enhancing children's WM and intelligence.

In our day-to-day experiences, we frequently encounter strong memory formation associated with highly positive or negative events. However, the precise mechanism underlying the encoding and integration of emotional stimuli into attention and WM remains uncertain. In this sense, Cianfanelli et al. conducted a study investigating the role of WM subcomponents in binding negative emotional and visuo-spatial information. By utilizing a dual task paradigm to interfere with the central executive (CE) subcomponent and an immediate post-task to interfere with the episodic buffer (EB) subcomponent, the study found that interference with the EB task prevented the emotion-enhancing effect of negative pictures, while interference with the CE did not. These findings highlight the key role of the EB and the involvement of pre-attentive automatic processes in binding emotional and visuo-spatial information. Similarly, Qiu et al. conducted a study focusing on the attentional capture of negative stimuli. By utilizing EEG recordings and machine learning techniques, the study revealed that visual awareness is crucial for the spatial attentional capture of fearful faces. Additionally, the researchers found that the fear-related effect persists and can modulate neural processes involved in subsequent cued spatial targets but also requires top-down attention to the faces. Furthermore, Qu et al. conducted a study to examine the impact of dynamic and static angry facial expressions on time perception. Their findings revealed that static angry faces had an earlier impact, leading to an overestimation of time due to early emotional arousal and attentional bias. In contrast, dynamic angry faces appeared to influence time perception by eliciting response inhibition and late sustained attention.

Transitions of consciousness are closely intertwined with our attention and WM, and they can also be influenced by emotions. However, comprehending the nature of consciousness poses a significant challenge. To delve into this enigmatic phenomenon, Quettier et al. conducted a study utilizing a binocular rivalry paradigm and a joystick to map the dynamics of conscious experience in response to faces displaying different emotions or genders. The results showed that formation was slower than dissolution in general. Additionally, participants preferred happy faces in emotion rivalry and their contents were slower to form and dissolve compared to neutral faces.

Rewards often lead to pleasure and positive feelings, and different perspectives on rewards can influence our cognitive function as well. In a study conducted by Giuffrida et al., they explored how different reward perspectives can influence participants' adaptation strategies by manipulating the reward regimen in a virtual competition. The findings indicate that participants do utilize different strategies based on the value of the reward, as evidenced by variations in response speed across different conditions. Although in some cases, participants may concurrently adjust their inhibition strategies as well, there is no significant difference in the duration of the inhibition process.

Understanding the interplay between attention, WM, and emotion also provides valuable insights for the application and user-friendly design. For instance, Zhang et al. conducted a study utilizing behavioral measures and eye-tracking techniques to investigate the impact of the layout of mobile map navigation icons on users' visual search efficiency. The results demonstrated that navigation icons employing color for layout coding exhibited the highest visual search efficiency and provided a superior user experience compared to other types of layouts. The researchers also recommended developers consider implementing regular color distribution and a larger area of the same color to enhance the overall user experience. By employing EEG technique, Chen J. et al. studied how pattern, lightness, and color factors of ceramic tiles influence customers' preferences. The findings revealed that light-toned tiles captured greater attention during the initial stages of visual processing. Subsequently, the patterned and neutral-colored tiles exhibited their impact during the mid-stage processing. Moreover, Qin et al. investigated the neural mechanism involved in recognizing graphic artifacts with varying degrees of repetition within a disorganized environment. The results revealed that the arrangement of the repeating graphics had a more pronounced effect on the later stages of cognitive processing, rather than on the earlier attentional features during the initial stages. Furthermore, by recording EEG data from individuals exposed to various price variances among purchase channels, Han and Zhang established a connection between potential neural indicators (i.e., N2 and P3) and customers' identification and attention distribution when evaluating product price variances across different purchase channels. These findings offer valuable implications and suggestions for economists and marketers within the relevant industry, providing insights on how to optimize strategies and improve customer experiences.

Aligned with our initial objective, this Research Topic brings together researchers from diverse disciplines focusing on visual attention, WM, and emotions. By exploring the complex interplay and mutual influence among these cognitive processes, our Research Topic not only provides fundamental insights into the interconnected nature of these three important processes but also extends its impact to other cognitive functions. Furthermore, it sheds light on various application fields, offering potential for optimizing commercial efficiency and enhancing overall human wellbeing. We firmly believe that this Research Topic holds great promise as a gateway for future research and calls for multidisciplinary efforts to further advance this field.

## Author contributions

All authors listed have made a substantial, direct, and intellectual contribution to the work and approved it for publication.

